# Real-world management of T1 high-grade bladder cancer: A 14-year retrospective single-center study

**DOI:** 10.14440/bladder.0039

**Published:** 2025-08-22

**Authors:** Zexuan Lv, Yi Feng, Bingyang Guo, Bin Jiang, Hongyu Zhang, Yin Lu, Wenfeng Gao, Jinlu Tang, Qing Ai, Qiang Cheng, HongZhao Li

**Affiliations:** 1 Department of Urology, The Third Medical Center, People’s Liberation Army General Hospital, Beijing 100039, China; 2 Department of Urology, Chinese People’s Liberation Army General Hospital, Beijing 100039, China; 3 School of Medicine, Nankai University, Tianjin 300071, China; 4 Chinese People’s Liberation Army Medical School, Beijing 100853, China

**Keywords:** Non-muscle invasive bladder cancer, Bladder preservation, Cystectomy, Bacillus Calmette–Guérin treatment, Tumor recurrence

## Abstract

**Background:**

T1 high-grade (T1HG) bladder cancer (BC) carries substantial risks of recurrence and progression, deserving of heightened clinical attention.

**Objective:**

The present study evaluated the management approaches and reported 14-year real-world outcomes in patients with T1HG BC from a single-center cohort.

**Methods:**

Data were retrospectively collected from primary T1HG patients who had undergone transurethral resection of bladder tumors (TURBT) at our institution between 2010 and 2023. A total of 165 patients were included. Their baseline characteristics, pathological findings, adjuvant therapies, recurrence, progression, and survival outcomes were analyzed. Predictors of tumor recurrence were modeled using multivariable analyses.

**Results:**

Tumor recurrence was significantly associated with post-operative Bacillus Calmette–Guérin (BCG) treatment (odds ratio [OR]: 0.315, *p*=0.001) and tumor multifocality (OR: 0.476, *p*=0.033). Among patients having received post-operative BCG treatment, tumor recurrence bore a significant correlation with tumor multifocality (OR: 0.328, *p*=0.027), and elevated body mass index (BMI) was identified as a potential accelerator of recurrence (hazard ratio: 1.098, *p*=0.01). The 10-year recurrence-free survival rate among all patients stood at 54.9% (95% confidence interval [CI]: 44.3–65.5%), with a median of 134 months (95% CI: 64.7–203.3 months). The rate of re-TURBT was 20%. The 10-year progression-free survival was 87.2% (95% CI: 81.0–93.5%) and the 10-year overall survival was 66.7% (95% CI: 54.0–79.4%). The 10-year cancer-specific survival and the 10-year cystectomy-free survival (CFS) rates were 93.7% (95% CI: 88.4–99.0%) and 86.3% (95% CI: 79.8–92.8%), respectively. Notably, BCG treatment significantly improved CFS (*p*=0.01).

**Conclusion:**

Recurrence in T1HG disease is associated with BCG therapy and tumor multifocality, with a high BMI potentially promoting relapse.

## 1. Introduction

According to the World Health Organization, bladder cancer (BC) represents one of the most common malignant tumors of the urinary tract. In 2020, over 500,000 new cases of BC were diagnosed globally, and this number is projected to exceed one million by 2040.^1^ As a high-grade, non-muscle-invasive malignancy, T1 high-grade (T1HG) BC is associated with a substantial risk of progression and aggressive biological behaviors. Clinically, its clinical management is notoriously challenging, since tumor cells at the T1 stage possess greater invasiveness compared to their counterparts at the Ta stage. Progression to the T2 stage significantly increases the risk of recurrence, the likelihood of necessitating cystectomy, and the potential for distant metastasis.^2^,^3^

The distinct molecular and biological features of T1HG BC play a critical role in its malignancy and recurrence risk. Studies have demonstrated that common genetic alterations, including *TP53* mutations and epigenetic modifications, significantly contribute to the aggressive behavior of the tumor and promote the infiltration of malignant cells into the bladder submucosa.^4^ In addition, immune escape mechanisms within the tumor microenvironment are also culpable for an increased risk of recurrence, despite early treatment.^5^ Multiple factors, including the tumor’s biological characteristics, the immune escape mechanisms within the tumor microenvironment, and some patient-specific factors, contribute to recurrence.^6^,^7^

One of the primary causes of local tumor recurrence is immune escape. Studies have shown that the immune environment of T1HG BC is characterized by immunosuppression mediated by tumor-associated macrophages and reduced T-cell activity, which allows the tumor to evade host immune surveillance.^8^ Surveillance for recurrence is essential for improving the prognosis of T1HG BC. Urine cytology and cystoscopy are the most commonly used methods for monitoring recurrence.^9^,^10^

Given the features of T1HG BC, this study aimed to retrospectively analyze single-center cases to evaluate the disease’s prognosis and current treatment landscape, to provide clinical insights into its diagnosis and treatment.

## 2. Methodology

### 2.1. Patient data

Included in this single-center retrospective study were patients who had undergone transurethral resection of bladder tumors (TURBT) at our institution between 2010 and 2023 and received a conclusive post-operative pathological diagnosis of T1HG BC. We evaluated their oncological outcomes, treatment options, and clinicopathological characteristics. The inclusion criteria were patients with primary BC who were hospitalized for TURBT at our hospital and postoperatively diagnosed as having T1HG BC. The ethics committee approved the study, and all patients provided informed consent. The procedures were performed by senior urologists, and at least two pathologists assessed post-operative pathological results.

We collected data on gender, age, comorbidities, such as hypertension and diabetes mellitus, body mass index (BMI), anesthesia modality, tumor multifocality, tumor size, pathological subtypes, presence of concomitant carcinoma *in situ*, choroidal infiltration, and Bacillus Calmette–Guérin (BCG) treatment. The primary outcome measures included recurrence-free survival (RFS), progression-free survival (PFS), overall survival (OS), cancer-specific survival (CSS), and cystectomy-free survival (CFS). For patients suffering from multiple relapses, the first relapse was used as the reference point, and the time of the first relapse was recorded. Other relevant data and follow-up information were harvested from each patient. After conventional TURBT, patients received intravesical induction therapy or other treatments, which were categorized into four regimens: (i) Intravesical BCG therapy, (ii) intravesical therapy with epirubicin or pirarubicin, (iii) other treatments, including chemotherapy, and (iv) no treatment.

### 2.2. Outcome measurement

Survival probabilities as the outcome metrics were calculated using the Kaplan–Meier method and presented as estimates and 95% confidence intervals (CIs). RFS was defined as the time from the initiation of surgical treatment to tumor recurrence, with the last disease assessment serving as the dropout time for patients who did not experience recurrence. PFS was defined as the time from surgery to tumor progression, with the last disease assessment as the dropout time for patients who did not experience progression. OS, excluding patients still alive at the last follow-up, was defined as the time from the start of surgical treatment to death from any cause. CFS was defined as the time from the start of treatment or the first dose of a therapeutic agent to the date of cystectomy or death due to any cause. In contrast, CSS was defined as the time from surgical treatment to death from BC, excluding patients who were still alive at the last follow-up.

Univariate analysis was conducted using the independent samples *t*-test for continuous variables and the Chi-square test for categorical data to evaluate the association between potential factors and tumor recurrence. Logistic regression was applied for multivariable analysis to assess the impact of multiple factors on tumor recurrence. In addition, the conditional Cox regression model was performed using the Prentice–Williams–Peterson framework. Survival analysis plots were generated using Prism 10.1.2, and all statistical tests were two-sided, with significance evaluated at 5% (*p*<0.05) using the Statistical Package for Social Sciences software (version 27.0).

## 3. Results

### 3.1. Baseline and perioperative information

A total of 165 patients were included in this study, with a follow-up cutoff date of January 2025. The cohort comprised 132 males (80%) and 33 females (20%), with a mean age of 67.5 ± 9.9 years and a mean BMI of 24.8 ± 3.4 kg/m^2^. Comorbidities included hypertension in 79 patients (47.9%) and diabetes mellitus in 28 patients (17%). The majority of patients were classified as American Society of Anesthesiologists (ASA) grade 2 (*n* = 130, 78.8%), with smaller proportions rated grade 1 (*n* = 4, 2.4%) and grade 3 (*n* = 31, 18.8%) (Table 1). Among the included patients, 122 (73.9%) underwent TURBT under general anesthesia, whereas 43 (26.1%) received lumbar or epidural anesthesia. A total of 86 patients (52.1%) presented with a solitary tumor, whereas 79 patients (47.9%) had multifocal tumors. The average tumor size (maximum diameter) of all patients measured 2.2 ± 1.0 cm. Pathological diagnoses revealed that 2 patients (1.2%) had concomitant carcinoma *in situ*, 5 patients (3%) had vascular invasion, 6 patients (3.6%) had urothelial carcinoma with squamous differentiation, 2 patients (1.2%) had partial micropapillary carcinoma, 1 patient (0.6%) had glandular differentiation, and 1 patient (0.6%) had neuroendocrine carcinoma. Postoperatively, 96 patients (58.2%) received regular BCG intravesical therapy, 29 (17.6%) were treated with pirarubicin infusion, 23 (14.0%) with epirubicin infusion, 11 (6.6%) received chemotherapy or other treatments, and 6 (3.6%) did not receive any adjuvant therapy. Post-operative recurrence occurred in 57 patients (34.5%), and progression was observed in 16 patients (9.7%). The median follow-up time lasted for 57 months (range: 12–179 months). Detailed information is provided in Table 2.

**Table 1:** Baseline information of all patients

Characteristics	Data
Number of patients	165 (100)
Sex	
Male	132 (80)
Female	33 (20)
Age, years (mean±SD)	67.5±9.9
BMI, kg/m^2^ (mean±SD)	24.8±3.4
Hypertension	
Yes	79 (47.9)
No	86 (52.1)
Diabetes	
Yes	28 (17)
No	137 (83)
ASA score	
1	4 (2.4)
2	130 (78.8)
3	31 (18.8)

Note: Data presented as *n* (%) unless stated otherwise.

Abbreviation: SD: Standard deviation.

**Table 2:** Perioperative information of all patients

Information	Data
Anesthesia method	
General anesthesia	122 (73.9)
Other	43 (26.1)
Tumor multifocality	
Solitary	86 (52.1)
Multifocal	79 (47.9)
Maximum diameter of the tumor, cm (mean±SD)	2.2±1.0
Concurrent carcinoma *in situ*	
Yes	2 (1.2)
No	163 (98.8)
Vascular infiltration	
Yes	5 (3.0)
No	160 (97.0)
Histological subtype	
Squamous differentiation	6 (3.6)
Partially micropapillary carcinoma	2 (1.2)
Glandular differentiation	1 (0.6)
Neuroendocrine carcinoma	1 (0.6)
Pure urothelial carcinoma	155 (93.9)
Post-operative adjuvant therapy	
Bacillus Calmette–Guérin	96 (58.2)
Pirarubicin	29 (17.6)
Epirubicin	23 (14.0)
Other	11 (6.6)
None	6 (3.6)

Note: Data are presented as *n* (%) unless stated otherwise.

Abbreviation: SD: Standard deviation.

### 3.2. Factors associated with tumor recurrence

Univariate, multivariate, and Cox regression analyses were performed on all patient samples. In contrast, only univariate and multivariate analyses were conducted for post-operative BCG-treated patients.

Univariate analysis was performed to evaluate potential predictors of tumor recurrence using categorical variables (Table 3) and continuous variables (Table 4) among the 165 patients. Tumor recurrence was found to be significantly correlated with age (*p*=0.039), tumor multifocality (*p*=0.014), and BCG treatment (*p*=0.001). No significant association was observed with gender, hypertension, diabetes mellitus, anesthesia mode, concurrent carcinoma *in situ*, pathological subtype, vascular infiltration, BMI, and tumor size.

**Table 3:** Univariate analysis of categorical variables of all patients

Variable	Group	Recurrence-free status		Chi-square	*p*-value

		No	Yes		
Sex	Female	22	11	0.027	0.521
	Male	86	46		
Hypertension	No	57	29	0.054	0.87
	Yes	51	28		
Diabetes	No	91	46	0.335	0.663
	Yes	17	11		
Anesthesia	General anesthesia	84	38	2.39	0.318
	Other	24	19		
Tumor multifocality	Solitary	64	22	6.383	0.014*
	Multifocal	44	35		
Concurrent carcinoma *in situ*	No	106	57	1.069	0.545
	Yes	2	0		
Pathological subtypes	No	101	54	0.097	1
	Yes	7	3		
Vascular infiltration	No	105	55	0.068	1
	Yes	3	2		
Bacillus Calmette–Guérin treatment	Yes	74	22	13.729	<0.001*
	No	34	35		

Note:

* Indicates statistically significant values at *p*<0.05.

**Table 4:** Continuous variable one-way analysis of variance of all patients

Variable	Mean	SD	Mean 95% CI		*t*	*p*-value

			Lower level	Upper level		
Age, years	66.98	9.92	65.37	68.59	4.311	0.039*
	72.31	8.00	68.05	76.57		
Body mass index, kg/m^2^	24.66	3.09	24.15	25.16	2.271	0.134
	26.00	5.46	23.09	28.91		
Maximum tumor diameter, cm	2.17	1.02	2.01	2.34	0.127	0.722
	2.27	1.04	1.71	2.82		

Note:

* Indicates statistically significant values at *p*<0.05.

Abbreviations: SD: Standard deviation; CI: Confidence interval.

Factors that showed significant associations with outcomes in the univariate analysis, including age, tumor focality, and BCG treatment, were subsequently included in the multivariate analysis for further evaluation (Table 5). The findings indicated that BCG treatment (odds ratio [OR] = 0.315, *p*=0.001) and tumor multifocality (OR = 0.476, *p*=0.033) were the critical factors linked to tumor recurrence. In other words, in patients with primary T1HG BC who underwent TURBT, tumor multifocality and BCG treatment were independent factors affecting tumor recurrence. In addition, post-operative BCG infusion therapy reduced the likelihood of recurrence compared to treatment without BCG, and recurrence was more common in patients with multifocal tumors than in those with a solitary tumor.

**Table 5:** Multifactorial analysis of all patients

Variable	β	*p*-value	Odds ratio	95% CI	

				Lower level	Upper level
Age	0.010	0.579	1.010	0.975	1.047
Tumor multifocality	−0.743	0.033*	0.476	0.240	0.943
Bacillus Calmette–Guérin treatment	−1.156	0.001*	0.315	0.158	0.627
Constant	−0.336	0.796	0.715	-	-

Note:

* Indicates statistically significant values at *p*<0.05.

Abbreviation: CI: Confidence interval.

Univariate analyses were conducted using categorical variables (Table 6) and continuous variables (Table 7) among the 96 patients who received post-operative BCG treatment, with tumor recurrence acting as the dependent variable, to further identify factors that were significantly associated with tumor recurrence in this subgroup. The analysis excluded patients who did not receive post-operative BCG treatment. The univariate analysis revealed a significant correlation between tumor recurrence and tumor multifocality (*p*=0.024) in this patient group. In addition, no significant correlation was revealed between tumor recurrence and sex, hypertension, diabetes, anesthesia modality, concurrent carcinoma *in situ*, pathological subtype, vascular invasion, age, BMI, or tumor size.

**Table 6:** Univariate analysis of categorical variables in patients treated with Bacillus Calmette–Guérin

Variable	Group	Recurrence-free survival status		Chi-square	*p*-value

		No	Yes		
Sex	Female	16	3	0.681	0.409
	Male	58	19		
Hypertension	No	39	12	0.023	0.879
	Yes	35	10		
Diabetes	No	61	18	0.004	0.947
	Yes	13	4		
Anesthesia	General anesthesia	60	17	0.155	0.694
	Other	14	5		
Tumor multifocality	Solitary	47	8	5.109	0.024*
	Multifocal	27	14		
Concurrent carcinoma *in situ*	No	72	22	0.607	0.436
	Yes	2	0		
Pathological subtypes	No	70	20	0.393	0.531
	Yes	4	2		
Vascular infiltration	No	72	21	0.19	0.663
	Yes	2	1		

Note:

* Indicates statistically significant values at *p*<0.05.

**Table 7:** One-way analysis of continuous variables of patients treated with Bacillus Calmette–Guérin

Variable	Mean	SD	Mean 95% CI		*t*	*p*-value

			Lower level	Upper level		
Age, years	65.64	9.12	63.52	67.75	0.588	0.445
	67.50	12.62	61.91	73.09		
Body mass index, kg/m^2^	24.68	3.18	23.94	25.41	2.919	0.091
	26.03	3.55	24.46	27.60		
Maximum tumor diameter, cm	2.06	0.97	1.83	2.28	0.038	0.846
	2.10	1.05	1.64	2.57		

Abbreviations: SD: Standard deviation; CI: Confidence interval.

Tumor multifocality, shown to bear a significant association with tumor recurrence by univariate analysis, was further subjected to multivariate analysis (Table 8). The multifactorial analysis revealed that tumor multifocality (OR = 0.328, *p*=0.027) remained a significant factor associated with tumor recurrence in patients who received post-operative BCG treatment. This suggests that tumor multifocality independently impacts recurrence risk in patients receiving post-operative BCG bladder perfusion. In addition, patients with multifocal tumors at diagnosis were more likely to develop recurrence than those with a solitary tumor.

**Table 8:** Multifactorial analysis of patients treated with Bacillus Calmette–Guérin

Variable	β	*p*-value	Odds ratio	95% CI	

				Lower level	Upper level
Tumor multifocality	−1.114	0.027*	0.328	0.122	0.883
Constant	−0.657	0.046	0.519	-	-

Note:

* Indicates statistically significant values at *p*<0.05.

Abbreviation: CI: Confidence interval.

### 3.3. Survival analysisπ

Cox regression analysis was conducted on all patient samples based on recurrence and time to recurrence for all of the factors above that may affect tumor recurrence (Table 9). The Cox regression analysis revealed that tumor recurrence was significantly correlated with tumor singularity and multifocality (hazard ratio [HR] = 0.477, *p*=0.012), BCG treatment (HR = 0.443, *p*=0.001), and BMI (HR = 1.098, *p*=0.01). In contrast, it was not significantly correlated with gender, age, hypertension, diabetes mellitus, anesthesia modality, tumor size, ASA grades, concurrent carcinoma *in situ*, pathological subtype, and vascular infiltration. Based on the results of the analysis, we were led to hypothesize that BCG treatment and tumor multifocality independently affect tumor recurrence. Solitary tumors are less likely to recur than multifocal ones, and BCG therapy is associated with a lower recurrence rate than non-BCG treatment. In addition, Cox regression results suggest that while an increase in BMI may accelerate the recurrence process, it may not be a dictating factor in tumor recurrence.

**Table 9:** Cox regression analysis of all patients

Variable	β	*p*-value	Hazard ratio	95% CI	

				Lower level	Upper level
Sex	0.103	0.778	1.108	0.543	2.262
Age	0.013	0.438	1.013	0.981	1.046
BMI	0.093	0.010*	1.098	1.023	1.179
Hypertension	0.015	0.957	1.015	0.565	1.716
Diabetes	0.117	0.746	1.124	0.553	2.286
Anesthesia	−0.137	0.661	0.872	0.473	1.607
Tumor multifocality	−0.741	0.012*	0.477	0.267	0.85
Maximum tumor diameter	−0.056	0.695	0.946	0.716	1.249
ASA score	-	0.981	-	-	-
Score 1	0.027	0.980	1.028	0.125	1.931
Score 2	0.072	0.844	1.074	0.526	2.193
Pathological subtypes	0.162	0.796	1.176	0.344	4.023
Vascular infiltration	−0.6	0.438	0.549	0.120	2.498
Bacillus Calmette–Guérin treatment	−0.814	0.010*	0.443	0.239	0.820

Note:

* Indicates statistically significant values at *p*<0.05.

Abbreviations: CI: Confidence interval.

#### 3.3.1. RFS

**Figure 1:**
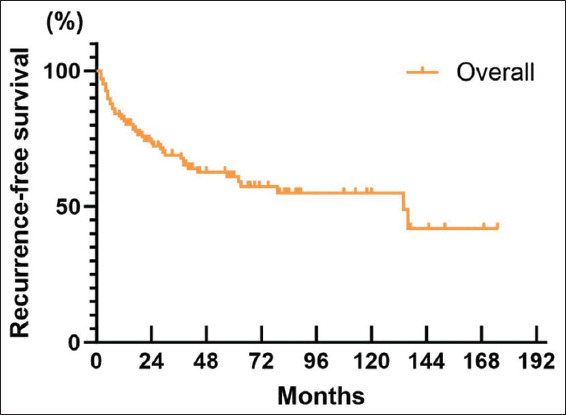
Overall patient recurrence-free survival curve

**Figure 2:**
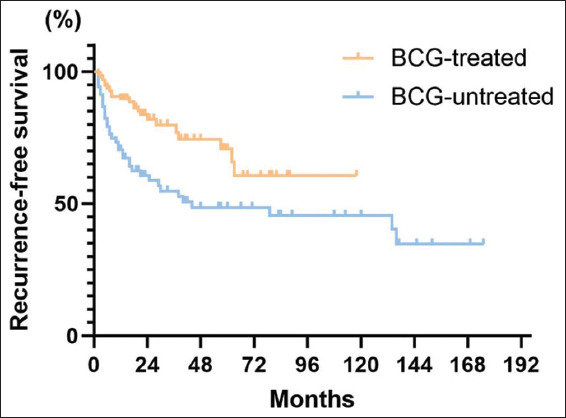
Recurrence-free survival curves grouped by treatment with or without Bacillus Calmette–Guérin

A total of 57 patients (34.5%) experienced recurrence, and 33 patients underwent re-TURBT, with a re-TURBT rate of 20%. The median RFS was 134 months (95% CI: 64.7–203.3), the 5-year RFS was 61.1% (95% CI: 52.1–70.1%), and the 10-year RFS was 54.9% (95% CI: 44.3–65.5%) for all patients (Figure 1). The 5-year RFS for patients who received post-operative BCG treatment was 65.7% (95% CI: 50.6–80.8%) with an unquoted median RFS, while among patients who did not get BCG treatment, the equivalent 5-year RFS was 48.5% (95% CI: 35.6–61.4%) with a median RFS of 44 months (95% CI: 0–126.9) (Figure 2). In addition, the administration of BCG exerted a statistically significant impact on patients’ recurrence (*p*=0.0027).

#### 3.3.2. PFS

**Figure 3:**
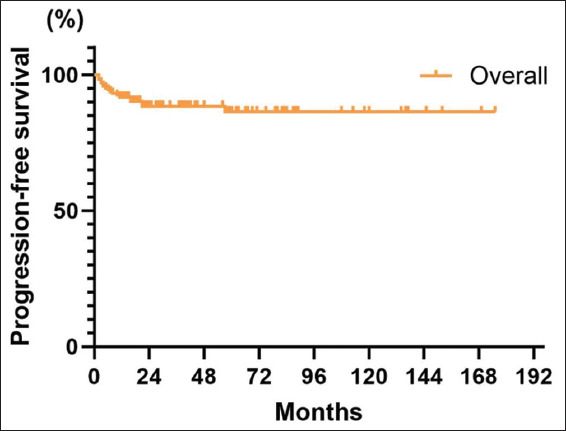
Overall patient progression-free survival curve

**Figure 4:**
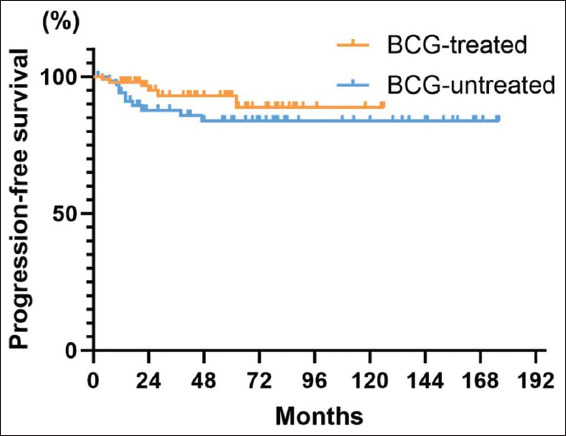
Progression-free survival curves grouped by treatment with or without Bacillus Calmette–Guérin

Disease progression (pT ≥ 2) took place in 16 (9.7%) of all patients, of which two (1.2%) developed distant metastasis. The 5-year PFS among all patients was 88.7% (95% CI: 83.0%–94.4%), and the 10-year PFS was 87.2% (95% CI: 81.0%–93.5%) (Figure 3). The 5-year PFS of patients treated with BCG after surgery was 92.9% (95% CI: 86.6–99.2%), whereas it was 83.7% (95% CI: 74.2–93.1%) in patients not treated with BCG (Figure 4). The impact of BCG treatment on patients’ disease progression was not statistically significant (*p*=0.15).

#### 3.3.3. OS

**Figure 5:**
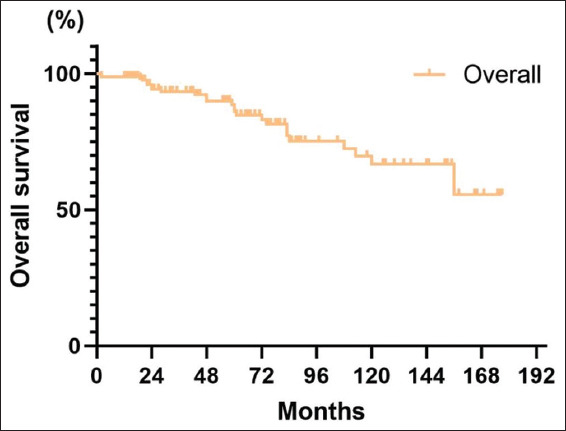
The overall patient survival curve

**Figure 6:**
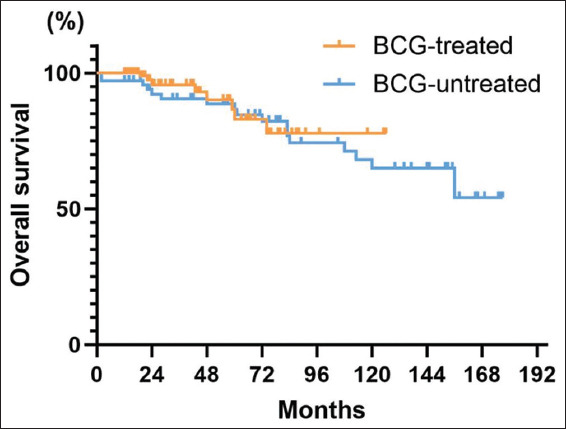
Overall survival curves grouped by treatment with or without Bacillus Calmette–Guérin

Among all patients, 26 (15.8%) were deceased. The 5-year and 10-year OSs were 85.9% (95% CI: 79.0–92.8%) and 66.7% (95% CI: 54.0–79.4%), respectively (Figure 5). Following surgery, the 5-year OS for patients treated with BCG was 83% (95% CI: 70.5–95.5%), while for patients not treated with BCG, it was 86.6% (95% CI: 77.8–95.4%) (Figure 6). Furthermore, BCG treatment had no statistically significant difference in patient survival or mortality (*p*=0.63).

#### 3.3.4. CSS

**Figure 7:**
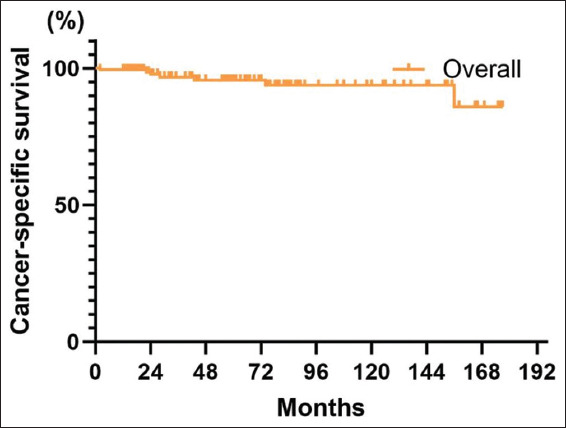
Overall patient cancer-specific survival curve

**Figure 8:**
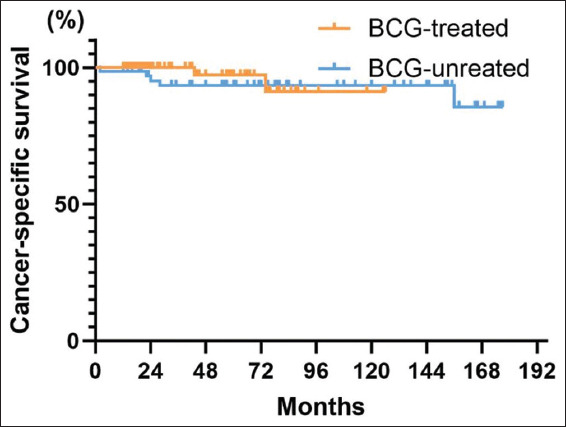
Cancer-specific survival curves grouped by treatment with or without Bacillus Calmette–Guérin

Seven patients (4.2%) succumbed to death due to BC caused by the disease. The 5-year CSS for all patients was 95.6% (95% CI: 91.7–99.5%), and the 10-year CSS was 93.7% (95% CI: 88.4–99.0%) (Figure 7). The 5-year CSS for the BCG-treated group was 97.2% (95% CI: 92–100%). In contrast, the 5-year CSS for the non-BCG-treated group was 93.3% (95% CI: 87.0–99.5%) (Figure 8). In addition, BCG treatment had no statistically significant impact on the CSS of patients (*p*=0.48).

#### 3.3.5. CFS

**Figure 9:**
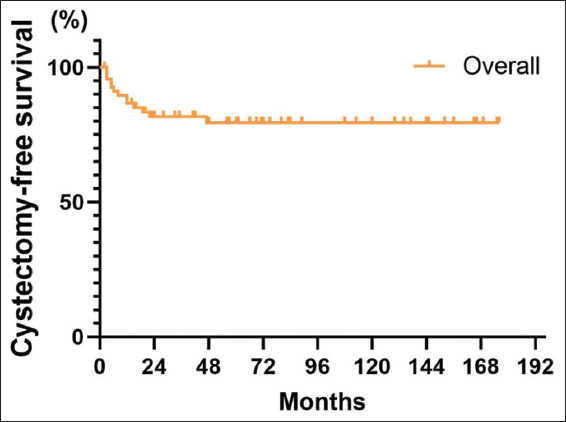
Overall patient cystectomy-free survival curve

**Figure 10:**
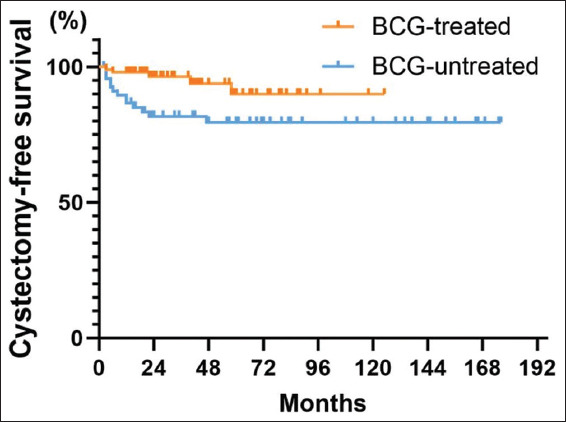
Cystectomy-free survival curves grouped by treatment with or without Bacillus Calmette–Guérin

Some patients underwent radical cystectomy due to recurrence and progression of the tumor, multiple recurrent recurrences, or prophylactic treatment. A total of 18 patients (10.9%) underwent radical cystectomy, and the 5-year, as well as 10-year CFS for the overall patient population, was 86.3% (95% CI: 79.8–92.8%) (Figure 9). The 5-year CFS was 90% (95% CI: 80.4–99.6%) in patients treated with BCG, and 79.5% (95% CI: 69.6–89.6%) in patients without receiving BCG (Figure 10). In addition, the impact of BCG use on patients’ CFS rate was statistically significant (*p*=0.01), showing that patients treated with BCG had a protracted post-operative CFS than those without receiving BCG.

## 4. Discussion

Compared to other studies, the strength of this study lies in its longer follow-up period and more systematic and accurate tracking of outcomes. The collection of potential recurrence-related indicators in this study differs from that used by other studies and is more comprehensive and precise. Although most of these indicators did not show significant associations with recurrence, their evaluation remains meaningful. Data analysis revealed a 5-year RFS of 61.1%, a 10-year RFS rate of 54.9%, and a median RFS of 134 months, exhibiting an improvement when compared to prior data.^11^,^12^ However, the 5-year and 10-year RFS rates were lower than PFS, OS, CSS, and CFS, highlighting that recurrence remains the most significant challenge in patients with T1HG BC. The risk of recurrence was highest among patients who underwent TURBT for bladder preservation. Nevertheless, the risk of disease progression and mortality remained low, with a 5-year PFS of 88.7% and a 10-year PFS of 90.0%. The 5-year OS was 85.9% and the 10-year OS was 66.7%. In addition, the 5-year and 10-year PFS were 87.2% and 87.2%, respectively. These rates indicate that OS was prolonged and the risk of progression was not high. The 5-year CSS and 10-year CSS were 95.6% and 93.7%, respectively, demonstrating that the specific fatality rate of T1HG BC is low, with patients more susceptible to recurrence than death caused by the disease. This aligns with findings from previous studies.^13^,^14^

Furthermore, patients treated with BCG bladder perfusion after surgery had a higher 5-year RFS, PFS, CSS, and CFS compared to those who did not receive BCG (including those treated with pirarubicin, epirubicin, other therapies, or no specific treatment), but exhibited a lower 5-year OS. The use of BCG could significantly improve RFS and CFS, but no significant improvements were observed in the other indicators. This suggests that BCG treatment reduces recurrence rates, lengthens RFS, and extends the time patients can retain their bladders. The significant effects of BCG on RFS and CFS emphasize its role in lowering the risk of radical cystectomy, which improves bladder preservation. The efficacy of BCG in reducing recurrence and conserving the bladder has been well-established in previous studies, which confirmed that BCG therapy is a viable option for bladder preservation. Even in older patients, BCG treatment significantly lowers recurrence and cancer-specific mortality rates in T1HG BC.^15^,^16^

The factors influencing the recurrence of T1HG BC remain a subject of ongoing debate. In this study, we identified and examined several factors potentially associated with tumor recurrence using multivariate analyses (univariate, multivariate, and Cox regression). BCG treatment significantly reduced the recurrence rate of T1HG tumors; tumors with multifocality were more likely to recur than those with a singular tumor. Among patients who received post-operative BCG treatment, tumor recurrence was associated with tumor multifocality. In addition, BCG treatment and tumor multifocality were independent factors affecting recurrence. We also found that an increase in BMI may accelerate tumor recurrence; however, it does not appear to be a decisive factor in determining recurrence. Further studies are needed to validate its overall impact on recurrence risk. In addition, literature has pointed out that in T1 BC patients treated with BCG, an increase in BMI is associated with a poorer prognosis, which is consistent with the findings of this study.^7^ In summary, tumor recurrence in patients with T1HG BC is strongly influenced by BCG therapy and tumor multifocality. In addition, several studies have highlighted the significant impact of neutrophil-to-lymphocyte ratio and basophil counts on recurrence, with higher neutrophil-to-lymphocyte ratio and basophil granulocyte counts correlating with poorer prognosis.^16^,^17^ Furthermore, research has documented that patients with type 2 diabetes mellitus, positive pre-operative urine cytology, and high CD163^+^ macrophage infiltration have an increased likelihood of recurrence and a worse prognosis.^18^-^20^

According to the European Association of Urology guidelines, cystoscopy and histological analysis of tissue obtained through TURBT are crucial for diagnosing T1HG BC and determining the appropriate treatment strategy. Patients are classified into low, intermediate, and high-risk groups based on their likelihood of recurrence and progression. A re-TURBT within 2–6 weeks is recommended if the initial resection is incomplete, no muscle tissue in the specimen, or high-grade or T1 tumors are identified. One immediate chemotherapy infusion is recommended for low-risk patients, whereas intermediate-risk patients should receive chemo-infusion or full-dose BCG intravesical immunotherapy for 1 year. For high-risk patients with BCG-refractory or BCG-non-responsive disease, full-dose intravesical BCG immunotherapy lasting for 1–3 years is recommended. BCG induction therapy followed by a maintenance regimen is advised for patients opting for bladder preservation. However, patients with multiple high-risk factors and BCG-refractory or BCG-naïve disease should be evaluated for cystectomy.^21^ The therapeutic management of T1HG BC has been summarized in several publications, which indicated that the current standard of care involves either radical cystectomy or bladder-sparing BCG induction and maintenance therapy.^10^,^22^,^23^ Moreover, intravesical instillation therapy with novel antibody-drug conjugates is emerging as a promising therapeutic modality.^24^

Several new approaches have shown significant promise in treating T1HG BC in recent years. For example, immune checkpoint inhibitors (ICIs) have been proven to be effective in treating T1HG BC and have found a wide array of applications. Zhou *et al*.^25^ developed and tested exosome-mimicking nanobubbles derived from macrophages as nanoplatforms for delivering ICIs in a mouse BC model. Their study found that this nanomedicine system improved precision in targeting tumors, demonstrated high stability, and provided sufficient biosafety.^25^ Advancements have also been made in using lysogenic viruses to treat BC. For example, Hu *et al*.^26^ found that intravesical instillation of AxdAdB-3, a lysogenic adenovirus with an E1B-55KD deletion and mutated E1A, significantly slowed bladder tumor growth in an orthotopic model.^26^ Lichtenegger *et al*.^27^ demonstrated that intratumoral delivery of XVir-N-31, a YB-1-selective adenovirus, significantly inhibited tumor progression and induced a higher level of immunogenic cell death in bladder tumor cells compared to wild-type adenovirus. In addition to directly lysing tumor cells, lysogenic viruses can trigger systemic immune responses against tumors, which can be enhanced by combining them with ICIs. Furthermore, genetic engineering can modify lysogenic viruses to increase their immunogenicity and tumor specificity while reducing toxicity. These modifications enhance the virus’s potential for BC treatment, and lysogenic viruses are expected to play a significant role in future therapies.^26^,^28^

Nevertheless, this study has several limitations, including a small sample size that may not accurately represent the broader patient population, regional bias due to its single-center design, and the need for multicenter studies to confirm the generalizability of the findings. In addition, the reliance on retrospective electronic medical records for data collection may have led to incomplete or missing data, potentially affecting the accuracy of the results.

## 5. Conclusion

With regard to T1HG BC, recurrence is a pivotal concern, with disease progression and mortality representing lesser threats in comparison. Tumor recurrence in T1HG BC is associated with the application of BCG as a treatment modality and tumor multifocality. In patients who receive BCG treatment, recurrence is linked to tumor multifocality. In addition, an increase in BMI may accelerate the recurrence process. Future research should involve larger sample sizes, mechanistic studies, and multicenter collaborations to enable precise treatment. At present, TURBT combined with bladder-sparing BCG induction and maintenance therapy, or radical cystectomy, remains the standard of care.

## Data Availability

The data that support the findings of this study are available from the corresponding author upon reasonable request.
